# The GEMMA speech database: VCV and VCCV words for the acoustic analysis of consonants and lexical gemination in Italian

**DOI:** 10.1016/j.dib.2022.108373

**Published:** 2022-06-27

**Authors:** Maria-Gabriella Di Benedetto, Luca De Nardis

**Affiliations:** a Department of Information Engineering, Electronics and Telecommunications, Sapienza University of Rome, Via Eudossiana 18, Rome 00184, Italy; b Massachusetts Institute of Technology, Cambridge, Massachusetts 02139, USA

**Keywords:** Speech processing, Speech recognition, Lexical gemination, Italian

## Abstract

The GEMMA database consists of recordings of disyllabic words: vowel-consonant-vowel (VCV) for nongeminate cases and vowel-consonant-consonant-vowel (VCCV) for geminate cases. The consonants in the words are stops /b/, /d/, /g/, /p/, /t/, /k/, affricates /ts/, /dz/, /ʧ/, /ʤ/, fricatives /f/, /v/, /s/, /z/ (singleton only) and /ʃ/ (geminate only), nasals /m/, /n/ and /ɲ/ (geminate only), and liquids /l/, /r/ and /λ/ (geminate only). The database also includes recordings for glides (/j/, /w/). The vowels in the words are /a, i, u/; words are symmetric with respect to vowel.

Six native adult speakers of Standard Italian, raised and living in Rome, Italy, three female and three male, uttered the speech materials in three different recording sessions; three repetitions for each word per speaker were therefore collected.

The dataset also includes the durations of vowel and consonant segments for all cases where the consonant can be singleton vs. geminate (see [1] and [2]).

**Specifications Table**

**Table:** 

Subject	Signal Processing
Specific subject area	Acoustic analysis of the Italian language
Type of data	Waveform Audio File Format (WAV) files, Comma Separated Value (CSV) files
How data were acquired	Recorded using a SONY ECM 144 omnidirectional microphone in an Amplisilence recording booth by Amplifon. Digitized using the UNICE software produced by VECSYS and converted in WAV format using the sox utility
Data format	Raw, Analyzed
Parameters for data collection	Recordings were collected in a sound-treated room. In case of evident mispronunciations, the speaker was compelled to repeat the word. The distance of the mouth of the speaker from the microphone was monitored during the recording sessions and was kept at about 20 cm. Speakers were asked to maintain their natural speaking style in order to mitigate the impact of variations in emission levels and tempo.
Description of data collection	The entire set of words was recorded three times in three different recording sessions, leading to three repetitions for each word and for each speaker. The words to be pronounced were presented to the speakers on cards, and the order of presentation was randomized in each recording session.
Data source location	Institution: Sapienza University of Rome City/Town/Region: Rome Country: Italy Data were collected in the Speech Communication Laboratory of the DIET Department, located in Via Eudossiana 18, 00184, Rome, Italy. Latitude: 41.893762939686034, Longitude: 12.493808281027881.
Data accessibility	Repository name: GEMMA [3]. Data identification number (DOI): 10.17632/dm5n5dzrp2.1 Direct link to the dataset: https://data.mendeley.com/datasets/dm5n5dzrp2/1
Related research articles	M.-G. Di Benedetto, L. De Nardis, Consonant gemination in Italian: the nasal and liquid case, Speech Communication, Volume 133, October 2021, pp. 62-80. DOI: 10.1016/j.specom.2021.07.006. M.-G. Di Benedetto and L. De Nardis, ”Consonant gemination in Italian: The affricate and fricative case” Speech Communication, Volume 134, November 2021, pp. 86-108. doi:10.1016/j.specom.2021.07.005.

## Value of the Data

• The GEMMA database provides an exhaustive set of recordings of Italian consonants, uttered under controlled conditions, and allows for systematic acoustic analyses of Italian consonants and lexical gemination (a long standing research issue in the field of acoustic phonetics).
• The data are of interest to researchers in the fields of speech communication, speech processing, and speech recognition.
• The data can be used to investigate the impact of lexical gemination on time, frequency, and energy parameters, for different consonant classes, with the aim of identifying a set of reliable cues that characterize gemination across consonant classes.
• A better understanding of gemination may help designing automatic speech recognition systems for the Italian language, by providing the ground for reliable detection of geminated consonants. This is an important issue since gemination is contrastive in Italian, that is, geminating a consonant changes the meaning of words (for example *pala* (shovel) vs. *palla* (ball)). For an exhaustive discussion on the phenomenon of gemination see the research papers [1], [2] using the GEMMA database and [4], addressing both lexical and syntactic gemination in Italian.
• The choice of the Italian language does not limit the scope of the GEMMA database. The research work on lexical gemination made possible by the database will be relevant to all languages in which gemination occurs, including Greek, Arabic and Japanese. See [1] for a detailed discussion on gemination across languages.

## 1 Data Description

The database includes both raw data, consisting of audio recordings, and analysed data(durations of vowel and consonant segments for all cases where the consonant can be  singleton vs. geminate), stored in a set of Comma Separated Values (CSV) text files. Details on the two datasets are provided below; note that consonants and glides are labeled using the ARPABET phoneme notation in file names, for both audio recordings and CSV files.

### 1.1 Audio recordings

The GEMMA database consists of recordings of disyllabic words, i.e. vowel-consonant-vowel (VCV) in the nongeminate case and vowel-consonant-consonant-vowel (VCCV) in the geminate case. The consonants in the words are stops /b/, /d/, /g/, /p/, /t/, /k/, affricates /ts/, /dz/, /ʧ/, /ʤ/, fricatives /f/, /v/, /s/, /z/ (singleton only) and /ʃ/ (geminate only), nasals /m/, /n/ and /ɲ/ (geminate only), and liquids /l/, /r/ and /λ/ (geminate only). In addition, the database also includes recordings for glides (/j/, /w/). The vowels in the words are /a, i, u/, that is a subset of the Italian vowel set /a, e, ɛ, i, o, ɔ, u/. Words are symmetric with respect to vowel.

Each repetition of each utterance is stored in a separate WAV file. Each file contains samples of a 1-channel recording, represented as 16 bits signed integers, with sampling rate 10 kHz. The database is organized in six folders:

• Affricates
• Fricatives
• Liquids
• Nasals
• Stops
• Glides

Each folder is organized in six subfolders, one for each speaker:

• FS1 (Female Speaker 1)
• FS2 (Female Speaker 2)
• FS3 (Female Speaker 3)
• MS1 (Male Speaker 1)
• MS2 (Male Speaker 2)
• MS3 (Male Speaker 3)

Files in each subfolder are named as follows:

<UTTERANCE><REPETITION><SPEAKER>.wav (e.g. ASSA1MS1.wav, ASSA2MS1.wav, and so on).

The number of files for each consonant in each subfolder is equal to the product of number of repetitions (3) by number of vowels (3) by number of forms (1 if the consonant can only be either singleton or geminate, 2 otherwise). The total number of files in the subfolder corresponding to each speaker for each class of consonants, and for glides, is provided in Table 1.

**Table 1:** Audio files in the subfolder of a given speaker; (G) - geminate (VCCV) form only, (S) - singleton (VCV) form only, (N/A) - not applicable. Highlighted rows indicate that duration parameters measurements are present in the corresponding consonant class measurement file.

Class	Phoneme	Gemination forms	Number of files	Total number of files
Affricates	/ts/	2	18	72
	/dz/	2	18	
	/ʧ/	2	18	
	/ʤ/	2	18	

Fricatives	/f/	2	18	72
	/v/	2	18	
	/s/	2	18	
	/z/	1 (S)	9	
	/ʃ/	1 (G)	9	

Liquids	/l/	2	18	45
	/r/	2	18	
	/λ/	1 (G)	9	

Nasals	/m/	2	18	45
	/n/	2	18	
	/ɲ/	1 (G)	9	

Stops	/b/	2	18	108
	/d/	2	18	
	/g/	2	18	
	/p/	2	18	
	/t/	2	18	
	/k/	2	18	

Glides	/j/	1 (N/A)	9	18
	/w/	1 (N/A)	9	

### 1.2 Duration measurement files

The measurement CSV files contain the durations of vowel and consonant segments for all cases where the consonant can be  singleton vs. geminate . Note that these duration values were used in the statistical analyses presented in [1] and [2]. The set of consonants for which measurements are available is highlighted in blue in Table 1.

Durations were measured using the xkl software [5]. Segments and corresponding durations were labeled as follows (see [1] and [2] for details):

• pre-consonant vowel duration V1d;
• closure duration C1d (for affricates and stops only);
• burst duration Bd (for stops only);
• Voice Onset Time VOT (for stops only);
• frication duration C2d (for affricates only);
• consonant duration Cd (for affricates one has Cd=C1d+C2d, for stops one has Cd=C1d+Bd);
• post consonant vowel duration V2d;
• entire word duration Utd.

Measurements were used to compute the following ratios between durations:

• Cd/V1d;
• C1d/V1d (for affricates and stops only);
• C2d/V1d (for affricates only).

All of the above is available in CSV files provided as part of the GEMMA database, one for each consonant class. Each row in a CSV file provides the information for a corresponding word; the information is organized as follows:

• name of the file;
• vowel;
• consonant phoneme, represented using the ARPABET notation;
• form (singleton or geminate);
• speaker;
• repetition;
• duration data for the specific consonant class.

An example of the content of a CSV file is presented in Table 2, showing the first rows of the file for affricate consonants.

**Table 2:** Description of the content of a CSV file that provides segment durations and durational ratios.

File Name	Vowel	Phoneme	Form	Speaker	Repetition	V1d	C1d	C2d	V2d	Utd	Cd	Cd_V1d_ratio	C1d_V1d_ratio
ACHA1FS1.wav	A	CH	S	FS1	1	156.4	72.6	105.4	131.3	465.7	178	1.138	0.464
ACHA2FS1.wav	A	CH	S	FS1	2	156.9	73.6	115.4	125.9	471.8	189	1.204	0.469
ACHA3FS1.wav	A	CH	S	FS1	3	164.1	76.4	116.8	96.6	453.9	193.2	1.177	0.465
ACHA1FS2.wav	A	CH	S	FS2	1	181.7	21	116.5	134.1	453.3	137.5	0.757	0.116
ACHA2FS2.wav	A	CH	S	FS2	2	172.1	10.8	120.9	109.4	413.2	131.7	0.765	0.063

## 2 Experimental Design, Materials and Methods

Recording sessions were carried out in an Amplisilence recording booth by Amplifon, featuring internal sound absorbing panels to avoid voice reverberation, and characterized by an external noise reduction of about 30 dB at the frequencies of interest. The microphone was an omnidirectional, monophonic SONY ECM 144 Electret Condenser Microphone, with a flat frequency response up to 15 kHz and a sensitivity of -55.3 dBm/mbar, selected according to the guidelines provided in [6]. The microphone was connected to a KENWOOD KT-48L tape recorder without automatic volume adjustment, to prevent unpredictable gain variations during the recordings. The connection between microphone and tape recorder used the built-in connection panel available in the recording booth, allowing thus to keep the door of the booth closed during the recording sessions. Words were written on cards that were presented to the speaker by the operator through the glass window of the recording booth. The distance of the speaker’s lips from the microphone was monitored during the recording sessions and was kept at about 20 cm, by having the microphone hanging in front of the speaker at a height adjusted to match the height of speaker’s mouth. Six adult Italian native speakers, three women and three men, aged between twenty-four and fifty, participated in the recordings sessions. Speakers were selected to cover both young and mature ages, were pronunciation defectless, and free of evident dialectal inflexions. All speakers were raised and living in Rome (Italy). Previous studies [7,8] suggested in fact that the Roman accent, although distinctive, is phonologically close to Standard Italian: it shares with Standard Italian a same phoneme inventory and phonotactic rules and shows similar behavior with respect to consonant gemination, in particular when spoken by educated people [8], although, as also pointed out by Payne in [7], the concept of Standard Italian is somewhat idealized. As a matter of fact, a progressive standardization of the Italian language was observed in a recent and comprehensive study on gemination across regional variations of Italian [9]. Note that a recent Italian read speech database also made use of speakers from Rome [10], and led to new insights on lexical vs. syntactic gemination [4]. An extension of the study of Italian geminate consonants [1,2] to include dialects would require the creation of specific datasets for specific dialects, a topic that was beyond the scope of this work and may form the object of future investigations.

Recording sessions were supervised by an acoustically trained person, in charge of pointing out evident mispronunciations and prompting a new recording when needed. Speakers were asked to maintain their natural speaking style in order to mitigate the impact of variations in emission levels and tempo. The use of multiple repetitions helped mitigating the risk of biases in the recorded material; cards were shuffled after each recording session.

The recordings were digitized using the UNICE software produced by VECSYS, by first applying a low pass filter with cut off frequency set at 5 kHz and then sampling at 10 kHz; each sample was quantized with 16 bits. The original UNICE proprietary files were then converted into WAV files using the sox open source utility, in order to offer a wide access to the material.

## Ethics Statement

Informed consent was obtained from all subjects involved in the data measurement campaign. As no personal data is shared with the paper, ethics consent was not required.

The paper is not currently being considered for publication elsewhere.

## CRediT authorship contribution statement

**Maria-Gabriella Di Benedetto:** Writing – review & editing, Writing – original draft, Supervision, Conceptualization, Investigation, Methodology, Project administration. **Luca De Nardis:** Data curation, Writing – original draft, Writing – review & editing, Software, Visualization.

## Declaration of Competing Interest

The authors declare that they have no known competing financial interests or personal relationships which have, or could be perceived to have, influenced the work reported in this article.

## Data Availability

The GEMMA speech database: VCV and VCCV words for the acoustic analysis of consonants and lexical gemination in Italian (Mendeley Data).
